# Immune checkpoint inhibitor rechallenge in advanced or metastatic non-small cell lung cancer: a retrospective cohort study

**DOI:** 10.1007/s00432-021-03901-2

**Published:** 2022-01-04

**Authors:** Ziyi Xu, Xuezhi Hao, Ke Yang, Qi Wang, Jing Wang, Lin Lin, Fei Teng, Junling Li, Puyuan Xing

**Affiliations:** 1 Department of Medical Oncology, National Cancer Center/National Clinical Research Center for Cancer/Cancer Hospital, Chinese Academy of Medical Sciences and Peking Union Medical College, Number 17 Panjiayuan Nan Li, Chao Yang District, Beijing, 100021 China; 2 Department of Medical Oncology, Cancer Hospital of Huanxing, Beijing, China; 3 Department of Medical Oncology, Beijing Chaoyang Sanhuan Hospital, Beijing, China

**Keywords:** Non-small cell lung cancer, Immune checkpoint inhibitor, Immunotherapy rechallenge

## Abstract

**Purpose:**

After progression to immunotherapy, the standard of care for non-small cell lung cancer (NSCLC) was limited. Administration of the same or different immune checkpoint inhibitors (i.e., ICI rechallenge) may serve as a novel option. The present study aimed to evaluate the efficacy of ICI rechallenge for NSCLC and explore prognostic factors.

**Methods:**

In this retrospective cohort study, data of advanced or metastatic NSCLC patients rechallenged with ICI at the Cancer Hospital, Chinese Academy of Medical Sciences, and Peking Union Medical College between December 2018 and June 2021 were retrieved. Progression-free, overall survivals (PFS; OS), etc. were calculated. Subgroup analyses were conducted according to baseline characteristics, prior treatment results, etc. for prognostic factor exploration using the Cox model.

**Results:**

Forty patients were included. Median age was 59 years. Thirty-one (78%) were male. Twenty-seven (68%) were smokers. Adenocarcinoma (28 [70%]) was the major histological subtype. Median PFS of patients receiving initial ICI was 5.7 months. The most common rechallenge regimens were ICI plus chemotherapy and/or angiogenesis inhibitor (93%). Seventeen (43%) were rechallenged with another ICI. Median PFS for ICI rechallenge was 6.8 months (95% CI 5.8–7.8). OS was immature. Tendencies for longer PFS were observed in nonsmoker or patients with adenocarcinoma, response of stable/progressive disease in initial immunotherapy, or whose treatment lines prior to ICI rechallenge were one/two. However, all results of prognostic factors were nonsignificant.

**Conclusion:**

ICI rechallenge may be an option for NSCLC after progress to immunotherapy. Further studies to confirm the efficacy and investigate prognostic factors are warranted.

**Supplementary Information:**

The online version contains supplementary material available at 10.1007/s00432-021-03901-2.

## Introduction

Lung cancer is the leading cause of cancer deaths worldwide (Bray et al. 2018). Non-small cell lung cancer (NSCLC) is the major histological type, accounting for almost 85% of lung cancer cases (Chen et al. 2014). When diagnosed as NSCLC, nearly 70% patients were with advanced or metastatic disease (Molina et al. 2008). For advanced or metastatic NSCLC with negative driver gene, immune checkpoint inhibitors (ICI) with or without chemotherapy were recommended ("NCCN" 2021). However, after progression, the standard of care is only chemotherapy. Novel regimens are worthy to be explored.

After progression to ICI, administration of the same or different ICI (i.e., ICI rechallenge) may serve as a novel treatment option. Several secondary analyses of clinical trials and a case report have demonstrated the potential efficacy of ICI rechallenge in advanced melanoma (Beaver et al. 2018; Long et al. 2017) and renal cell carcinoma (Escudier et al. 2017; George et al. 2016; Rebuzzi et al. 2018). In terms of rechallenge with the same ICI (defined as the ICI used for rechallenge was the same as the one for the initial immunotherapy) in NSCLC, a retrospective analysis of the phase III OAK study indicated that patients received atezolizumab treatment beyond progression (TBP) had numerically longer median overall survival (OS) (Gandara et al. 2018). The OS in atezolizumab TBP arm, other anticancer treatment arm, and no anticancer treatment arm were 12.7 months vs 8.8 months vs 2.2 months, respectively. Four real-world studies in China, Europe, and USA (Ge et al. 2020; Metro et al. 2019; Ricciuti et al. 2019; Stinchcombe et al. 2020) and a case report (Ito et al. 2020) also showed promising antitumor activity of rechallenge with the same ICI. Although data of patients rechallenged with different ICI were limited, encouraging benefit was observed in a case series (Fujita et al. 2018). In the case series (Fujita et al. 2018), 12 patients previously treated with nivolumab were rechallenged with pembrolizumab. The median progression-free survival (PFS) was 3.1 months.

The present retrospective cohort study aimed to evaluate the efficacy of ICI rechallenge for NSCLC and explore prognostic factors. It provided new evidence of later-line treatment after progression to ICI.

## Methods

### Study design and patients

In this retrospective cohort study, advanced or metastatic NSCLC patients rechallenged with ICI (whether rechallenge with the same ICI or not) at the Cancer Hospital, Chinese Academy of Medical Sciences, and Peking Union Medical College between December 2018 and June 2021 were included for analysis. The present study was approved by the ethics committee of Cancer Hospital, Chinese Academy of Medical Sciences and Peking Union Medical College (approval number: 21/323-2994). Informed consent was waived by the ethics committee.

Patients aged 18–75 years; with advanced or metastatic NSCLC and at least target lesion; rechallenged with single-agent ICI or ICI plus chemotherapy and (or) angiogenesis inhibitor after initial immunotherapy were eligible. Only those who discontinued initial ICI due to disease progression and rechallenged were included. Those who rechallenged ICI after initial treatment discontinuation by adverse events, those who received initial ICI as adjuvant or maintenance therapy, and those had no response evaluation after ICI rechallenge were excluded.

### Study assessment

Demographic and baseline characteristics, and data of tumor treatment were retrieved from the health information system, including age, sex, smoking status, Eastern Cooperative Oncology Group performance status (ECOG PS), tumor TNM stage, histological subtype, data of initial immunotherapy and ICI rechallenge, etc.

Efficacy end points were PFS (defined as the time from study treatment initiation to disease progression or death from any cause) per Response Evaluation Criteria in Solid Tumours (RECIST) 1.1, OS (defined as the time from study treatment initiation to death from any cause), overall response rate (ORR; defined as the proportion of patients with complete response [CR] or partial response [PR]), and disease control rate (DCR; defined as the proportion of patients with CR, PR or stable disease [SD]) for ICI rechallenge.

### Statistical considerations

The continuous and categorical data were presented as medians [quartile 1 (Q1) and quartile 3 (Q3)] and numbers (percentages), respectively. Median PFS and OS and 95% confidence intervals (CI) were estimated using the Kaplan–Meier method.

Subgroup analyses for efficacy predictors were conducted based on smoking status (nonsmoker vs smoker), ECOG PS (≥ 2 vs 0–1), histological subtypes (squamous carcinoma vs adenocarcinoma), best overall response (BOR; SD/progressive disease [PD] vs CR/PR), treatment lines prior to ICI rechallenge (≥ three vs one/two), rechallenge with the same ICI (no vs yes), ICI rechallenge regimens (ICI plus chemotherapy vs ICI plus angiogenesis inhibitor vs ICI plus chemotherapy and angiogenesis inhibitor vs monotherapy), brain, liver, or bone metastases (yes vs no), programmed death ligand 1 (PD-L1) tumor proportion score (TPS; < 1% vs ≥ 1%), and positive driver genes (*EGFR* vs *KRAS* vs *HER2* vs wildtype). Hazard ratios (HR) and 95% CI were calculated using the Cox proportional hazard model. *P* < 0.05 was considered statistically significant. All statistical analyses were performed using SPSS v26.0 (IBM Corp., Armonk, NY, USA).

## Results

Forty patients rechallenged with ICI between December 2018 and June 2021 were included. Median follow-up was 8.0 months (IQR 7.9–8.5 months). Median age was 59 years (IQR 55–65 years). Thirty-one patients (78%) were male. Twenty-seven (68%) were smokers. Twenty-nine (73%) had ECOG PS of 0 or 1. Adenocarcinoma (28 [70%]) was the major histological subtype, and one adenosquamous carcinoma (3%) was also included. At diagnosis, most patients (29 [73%]) were at stage IV. Driver genes were tested in 30 patients, of which 17 (57%) were positive. PD-L1 data were available in 15 patients. Eight and seven (53%; 47%) were with PD-L1 TPS ≥ 1% and < 1%, respectively (Table 1).

**Table 1:** Demographic and baseline characteristics

	Results (*n* = 40)
Age, years	59 (IQR 55–65)
> 60	16 (40%)
≤ 60	24 (60%)
Sex	
Male	31 (78%)
Female	9 (23%)
Smoking status	
Smoker	27 (68%)
Nonsmoker	13 (33%)
ECOG PS at ICI rechallenge initiation	
0–1	29 (73%)
≥ 2	11 (28%)
Histological subtype	
Adenocarcinoma	28 (70%)
Squamous carcinoma	11 (28%)
Adenosquamous carcinoma	1 (3%)
Clinical stage at diagnosis	
I	4 (10%)
II	2 (5%)
III	5 (13%)
IV	29 (73%)
Metastatic sites at ICI rechallenge initiation	
Brain	10 (25%)
Liver	4 (10%)
Bone	6 (15%)
Driver genes	
Number tested	30 (75%)
*EGFR*-mutated	6/30 (20%)
*KRAS*-mutated	8/30 (27%)
*HER2*-mutated	3/30 (10%)
Wildtype	13/30 (43%)
Driver gene unknown	10 (25%)
PD-L1 TPS	
Number tested	15 (38%)
≥ 1%	8/15 (53%)
< 1%	7/15 (47%)
PD-L1 TPS unknown	25 (63%)

*ICI* immune checkpoint inhibitor, *PD-L1* programmed death ligand 1, *TPS* tumor proportion score

The percentages might not equal 100% on account of rounding. ECOG PS, Eastern Cooperative Oncology Group performance status

In reference to the initial immunotherapy, most patients (21 [53%]) had received immunotherapy plus chemotherapy. Median PFS was 5.7 months (95% CI 4.1–7.2 months). Fourteen (35%), nineteen (48%), and seven (18%) had achieved PR, SD, and PD, respectively. After progression to the first immunotherapy, the majority of patients (33 [83%]) were directly rechallenged with ICI. Three (8%) received targeted therapy and four (10%) received chemotherapy between two lines of immunotherapy. Treatment lines prior to ICI rechallenge were one in 17 patients (43%), two in 12 (30%), and ≥ three in 11 (28%). The most common rechallenge regimens were ICI plus chemotherapy and (or) angiogenesis inhibitor (37 [93%]). And 17 patients (43%) were rechallenged with another ICI (Table 2).

**Table 2:** Previous treatment and ICI regimens

	Results (*n* = 40)
Initial immunotherapy regimen	
Anti-PD-1 monotherapy	10 (25%)
Anti-PD-1 + chemotherapy	21 (53%)
Combined with paclitaxel/paclitaxel liposome/nab-Pacilitaxel ± carboplatin	12 (30%)
Combined with pemetrexed + carboplatin/cisplatin	8 (20%)
Combined with gemcitabine	1 (3%)
Anti-PD-1 + angiogenesis inhibitor	5 (13%)
Combined with anlotinib	3 (8%)
Combined with bevacizumab/apatinib	2 (5%)
Anti-PD-1 + chemotherapy + angiogenesis inhibitor	4 (10%)
Combined with pemetrexed ± carboplatin + bevacizumab	4 (10%)
Progression-free survival of initial immunotherapy, months	5.7 (95% CI 4.1–7.2)
Best overall response to initial immunotherapy	
Complete response	0
Partial response	14 (35%)
Stable disease	19 (48%)
Progressive disease	7 (18%)
Treatment between two lines of ICI	
Targeted therapy	3 (8%)
Chemotherapy	4 (10%)
No	33 (83%)
Treatment lines prior to ICI rechallenge	
1	17 (43%)
2	12 (30%)
≥ 3	11 (28%)
ICI rechallenge regimen	
Anti-PD-1 monotherapy	3 (8%)
Anti-PD-1/Anti-PD-L1 + chemotherapy	17 (43%)
Anti-PD-1 + nab-Pacilitaxel	11 (28%)
Anti-PD-1 + gemcitabine	2 (5%)
Anti-PD-1 + pemetrexed + cisplatin	1 (3%)
Anti-PD-1 + irinotican/vinorelbine	2 (5%)
Anti-PD-L1 + nab-Pacilitaxel	1 (3%)
Anti-PD-1 + angiogenesis inhibitor	10 (25%)
Combined with bevacizumab	1 (3%)
Combined with anlotinib	3 (8%)
Combined with apatinib	6 (15%)
Anti-PD-1 + chemotherapy + angiogenesis inhibitor	10 (25%)
Combined with nab-Pacilitaxel/paclitaxel liposome + bevacizumab/anlotinib	4 (10%)
Combined with pemetrexed + bevacizumab	3 (8%)
Combined with S-1/irinotican + anlotinib	2 (5%)
Combined with vinorelbine + apatinib	1 (3%)
Rechallenge with the same ICI^a^	
Yes	23 (58%)
No	17 (43%)

*ICI* immune checkpoint inhibitor

^a^Patients who progressed from initial ICI treatment were rechallenged with the same ICI as the initial one. The percentages might not equal 100% on account of rounding

**Fig. 1:**
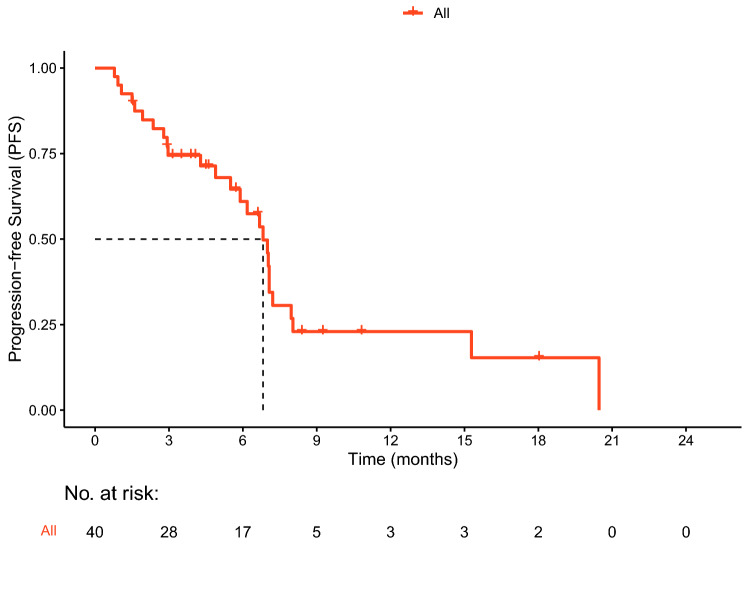
Kaplan–Meier curve of progression-free survival

During follow-up, 26 cases (65%) of progression occurred and eight patients (20%) died. Median PFS was 6.8 months (95% CI 5.8–7.8 months; Fig. 1). OS data were immature. Nine patients (22.5%) achieved PR. SD was observed in 25 cases (62.5%). ORR was 22.5% and DCR was 85% (Table 3).

**Table 3:** Response rate to ICI rechallenge

Overall best response	*n* (%)
CR	0
PR	9 (22.5%)
SD	25 (62.5%)
PD	6 (15.0%)
ORR	9 (22.5%)
DCR	34 (85.0%)

*n* number, *CR* complete remission, *PR* partial response, *SD* stable disease, *PD* progressive disease, *ORR* objective response rate (ORR = CR + PR), *DCR* disease control rate (DCR = CR + PR + SD)

**Fig. 2:**
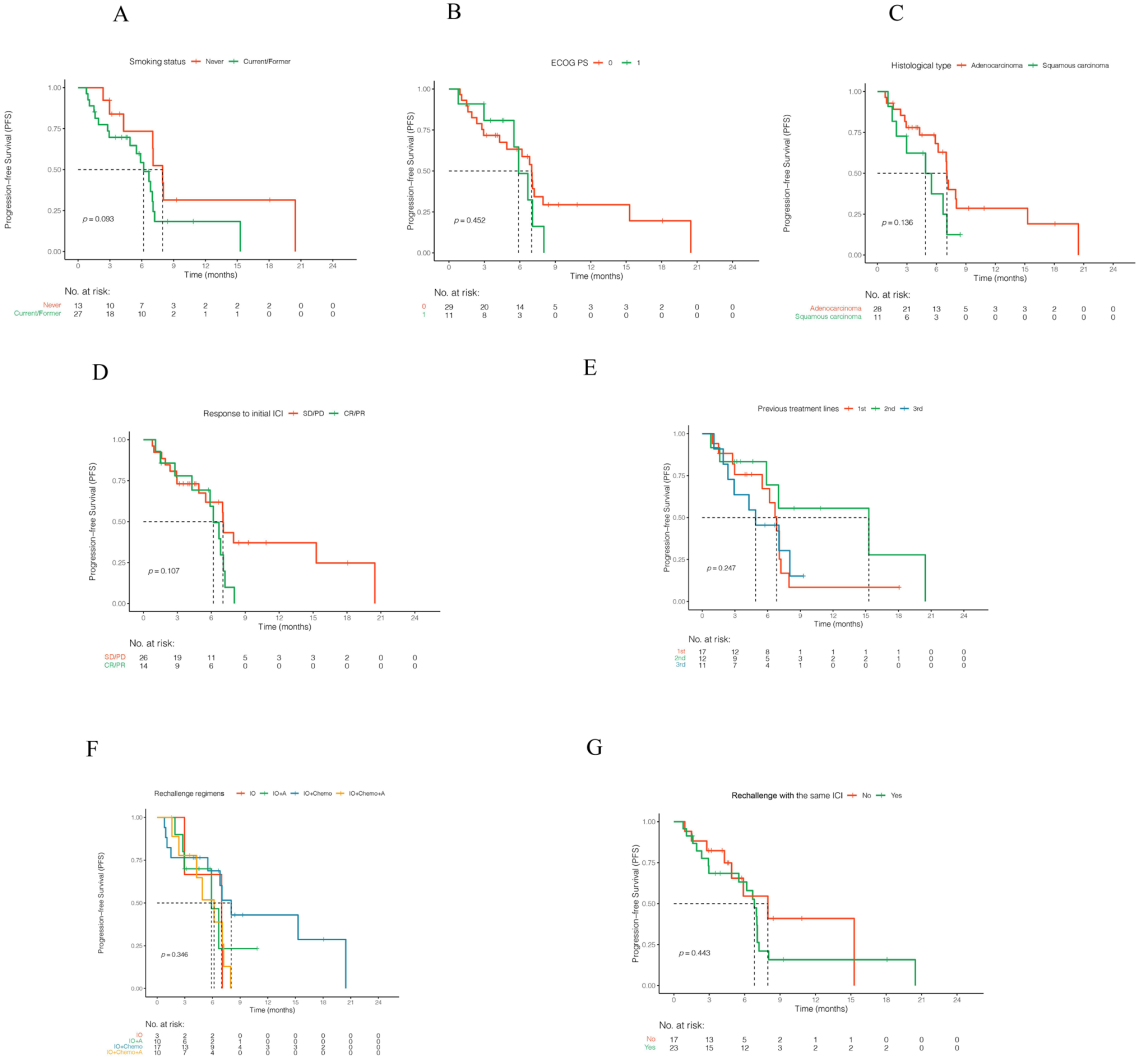
Kaplan–Meier curve of progression-free survival in patients with different smoking status (**A**), Eastern Cooperative Oncology Group performance status (**B**; ECOG PS), histological type (**C**), response to initial immunotherapy (**D**), previous treatment lines (**E**), rechallenge regimens (**F**), and the same immune checkpoint inhibitor rechallenge or not (**G**). *ICI* immune checkpoint inhibitor

**Fig. 3:**
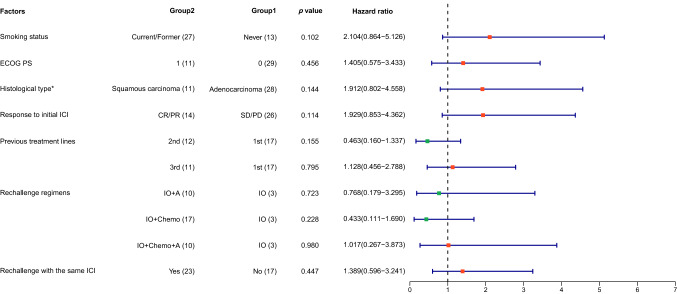
Forest plot of progression-free survival in patients with different smoking status, Eastern Cooperative Oncology Group performance status (ECOG PS), histological type, response to initial immunotherapy, previous treatment lines, rechallenge regimens, and the same immune checkpoint inhibitor rechallenge or not. ICI, immune checkpoint inhibitor. *One adenosquamous carcinoma was excluded from analyses

For subgroup analyses, tendencies for longer PFS were observed in nonsmoker or patients with adenocarcinoma, with BOR of SD/PD in initial immunotherapy, or whose treatment lines prior to ICI rechallenge were one/two. However, all HR between these subgroups were nonsignificant (Fig. 2 and 3). ECOG PS, rechallenge with the same ICI or not, ICI rechallenge regimens, metastatic sites, PD-L1 TPS, and driver genes did not affect PFS, either (Figs. 2, 3, S1 and S2).

## Discussion

Treatment after progression to ICI in NSCLC is limited. Some studies indicated ICI rechallenge might be a potential option (Fujita et al. 2018; Gandara et al. 2018; Metro et al. 2019; Ricciuti et al. 2019; Stinchcombe et al. 2020). Interestingly, in the present study, median PFS of initial immunotherapy was 5.8 months, while that of ICI rechallenge was 6.8 months. On one hand, the overall median PFS with initial immunotherapy may be influenced by some patients with early resistance. In our study, seven patients developed PD after only two cycles of immunotherapy-based therapy, which may be due to the early resistance to the combined agents rather than ICI. Previous studies showed a delayed onset of action of ICIs with a median time to response of 2.05–3.3 months (Chen et al. 2021; Hida et al. 2017; Rizvi et al. 2015). After re-administration of ICI and change of the combined regimens, these patients responded well. On the other hand, the contradictory results may be because of different ICI combination regimens in the two lines of immunotherapy. Compared with initial immunotherapy, ICI rechallenge regimens consisted of less monotherapy and ICI plus chemotherapy, and more ICI plus angiogenesis inhibitor with or without chemotherapy. ICI and anti-angiogenic agents have synergistic effect. As a critical angiogenic factor, vascular endothelial growth factor (VEGF) can repolarize tumor-associated macrophages to M2-like phenotypes (Fukumura et al. 2018), inhibit the maturation of dendritic cells (Gabrilovich et al. 1996), promote regulatory T-cell infiltration (Fukumura et al. 2018), and induce CD8^+^ T-cell exhaustion (Kim et al. 2019), and thus can lead to immune suppression and reduce effectiveness of ICI. Clinical studies also showed the efficacy of ICI plus VEGF inhibitors (Neal et al. 2020; Seto et al. 2020; Socinski et al. 2020; Zhou et al. 2020). Thus, additional VEGF inhibitor may bring benefit to ICI rechallenge. However, the findings should be confirmed by further studies.

Currently, the effectiveness of ICI rechallenge remains controversial. Some studies showed NSCLC diseases resistant to initial ICI therapies might display limited responses to ICI rechallenge, and might confer clinical benefits only in a small fraction, with the ORR of 0–8.5%, median PFS of 1.5–2.9 months, and median OS of 6.5–11.0 months (Fujita et al. 2019; Katayama et al. 2019; Teraoka et al. 2021; Watanabe et al. 2019); whereas in additional studies of ICI rechallenge, it was proposed as a potentially feasible option for those who suffered disease progression after initial ICI treatments, with the ORR of 11.6–23.0%, median PFS or duration of treatment of 4.1–9.1 months, and median OS of 9.5–26.6 months (Ge et al. 2020; Inno et al. 2021; Metro et al. 2019; Neal et al. 2020; Ricciuti et al. 2019; Stinchcombe et al. 2020). In the present study, OS data were immature and median follow-up was 8.0 months. Thus, median OS will be longer than 8.0 months. And, other efficacy end points of our study were in the range of the above-mentioned studies. Unlike the previous studies that mainly focused on ICI monotherapy rechallenge after ICI monotherapy (Fujita et al. 2018; Metro et al. 2019; Ricciuti et al. 2019; Stinchcombe et al. 2020), the majority of patients in our study received ICI combined with chemotherapy or anti-angiogenic agents as ICI rechallenge. In this context, the data from our study may provide some insights into future therapeutic strategies for advanced NSCLC.

Efficacy predictor analyses in our study showed no significant results. It may be attributed to the small sample size. The historical data on response to ICI rechallenge in different subgroups were limited. A retrospective cohort study (Ge et al. 2020) reported that males, squamous histology, no brain or liver metastases, any age, not beyond ≥ the third treatment line, with PR to the previous ICI, and monotherapy as previous ICI can benefit more from ICI rechallenge compared with other treatment. For initial immunotherapy, patients with smoking exposure (Kim et al. 2017; Zhao et al. 2021), better ECOG PS (Zhao et al. 2021), higher PD-L1 expression (Duchemann et al. 2021), and absence of liver metastasis (Zhao et al. 2021) benefited more from the treatment. Theoretically, previous treatment lines and response to initial immunotherapy can lead to various efficacy of ICI rechallenge. And rechallenge with the same ICI or not as well as rechallenge regimen may also have different antitumor activity. All above-mentioned potentially prognostic factors of ICI rechallenge should be explored in future prospective studies.

Four trials of ICI rechallenge for NSCLC are ongoing. Two single-arm, phase II trials (NCT04670913 (Xing et al. 2021) and NCT03689855) aimed to assess the efficacy of camrelizumab plus apatinib (VEGF receptor 2 TKI) and atezolizumab plus ramucirumab (anti-VEGF receptor 2 antibody). The remaining two randomized, controlled phase III trials were designed to compared efficacy and safety of atezolizumab plus cabozantinib (multitargeted TKI) vs docetaxel (NCT04471428), and pembrolizumab plus lenvatinib vs docetaxel plus lenvatinib (NCT03976375). The results will bring new evidence of ICI rechallenge for NSCLC.

There are several limitations in the present study. First, the biases were inevitable due to the retrospective nature of the study, including selection bias as ICI rechallenge was based on the physician's discretion. Second, the sample size was small and insufficient for efficacy predictor analyses.

In conclusion, the present study suggested that ICI rechallenge may serve as an option for NSCLC patients previously treated with immunotherapy. The efficacy should be confirmed in further investigations.

## Supplementary Information

Below is the link to the electronic supplementary material.

Supplementary file1 (DOCX 546 KB)

## Data Availability

Data and materials are available on request.
