# Supplementary material for: Immune checkpoint inhibitor rechallenge in advanced or metastatic non-small cell lung cancer: a retrospective cohort study
Source: J Cancer Res Clin Oncol. 2022 Jan 4;148(11):3081–9. doi: 10.1007/s00432-021-03901-2 (PMC9508034; doi:10.1007/s00432-021-03901-2)
Supplement: Supplementary file 1 — Supplementary file1 (DOCX 546 KB) [file 432_2021_3901_MOESM1_ESM.docx]

***Supplementary Material***

**
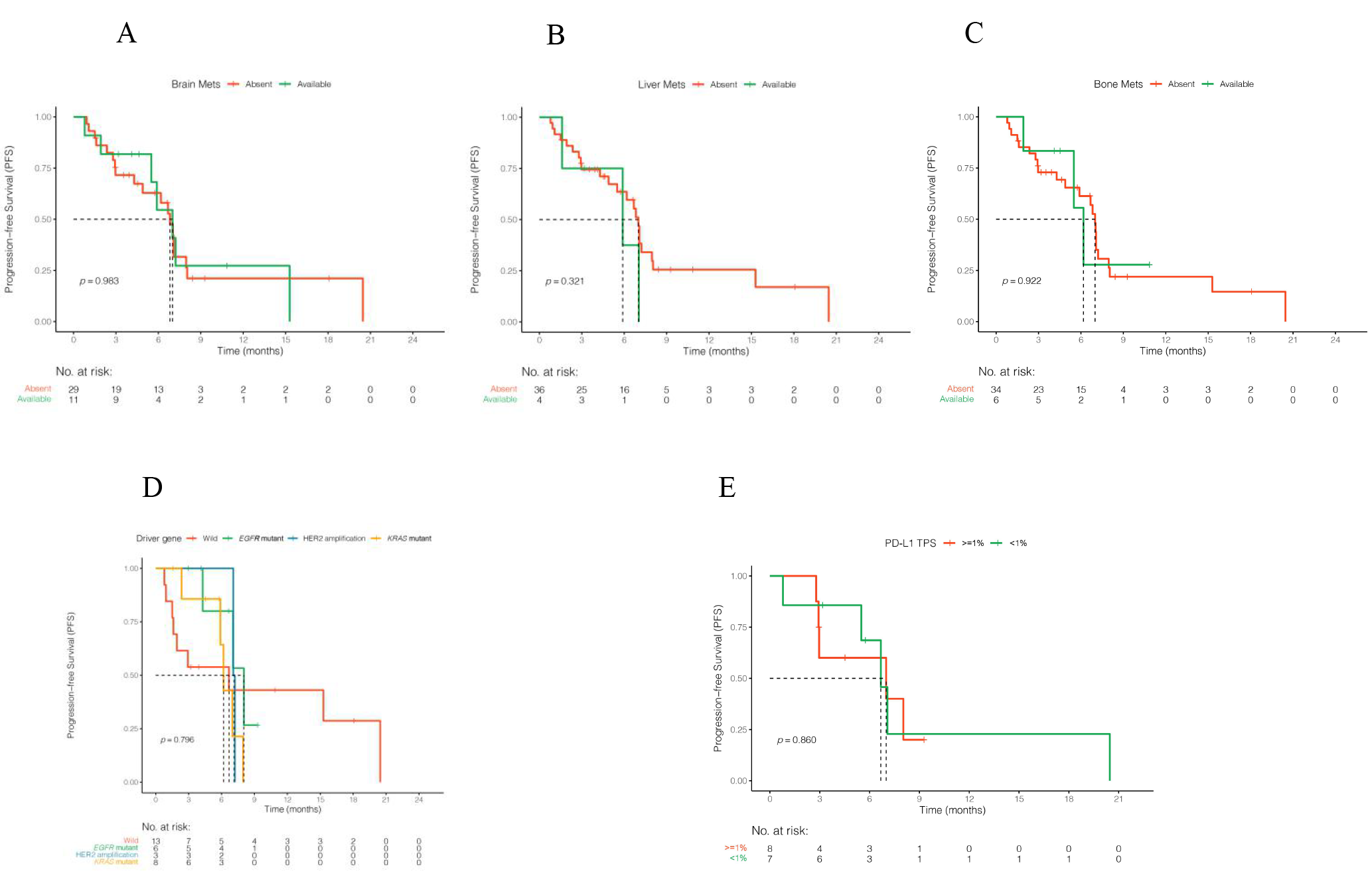
**

Figure S1. Kaplan-Meier curve of progression-free survival in patients with brain (A), liver (B), bone metastases (mets) or not (C), different driver genes (D), and programmed death ligand 1 (PD-L1) tumor proportion score (TPS; E).

**
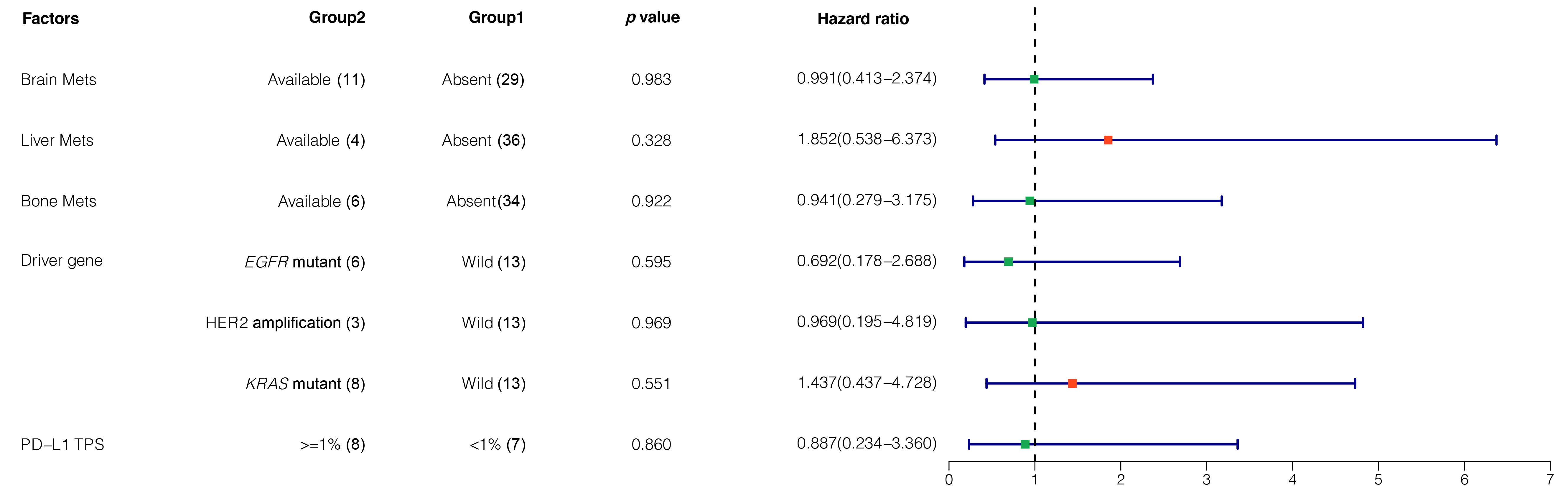
**

Figure S2. Forest plot of progression-free survival in patients with brain, liver, bone metastases (mets) or not, different driver genes, and programmed death ligand 1 (PD-L1) tumor proportion score (TPS).
